# Predictors of prolonged length of hospital stay and in-hospital mortality among adult patients admitted at the surgical ward of Jimma University medical center, Ethiopia: prospective observational study

**DOI:** 10.1186/s40545-020-00230-6

**Published:** 2020-06-16

**Authors:** Gosaye Mekonen Tefera, Beshadu Bedada Feyisa, Gurmu Tesfaye Umeta, Tsegaye Melaku Kebede

**Affiliations:** 1 Department of Pharmacy, Clinical Pharmacy Unit, Ambo University, Ambo, Ethiopia; 2 Department of Public Health, Nutrition Course Unit, Ambo University, Ambo, Ethiopia; 3 School of Pharmacy, Department of Clinical Pharmacy, Jimma University, Jimma, Ethiopia

**Keywords:** In-hospital mortality, Prolonged length of hospital stay, Predictors, Surgery, Ethiopia

## Abstract

**Background:**

Data regarding prolonged length of hospital stay (PLOS) and in-hospital mortality are paramount to evaluate efficiency and quality of surgical care as well as for rational resource utilization, allocation, and administration. Thus, PLOS and in-hospital mortality have been used as a surrogate indicator of satisfactory treatment outcome and efficient utilization of resources for a given health institution. However, there was a scarcity of data regarding these issues in Ethiopia. Therefore, this study aimed to assess treatment outcome, length of hospital stay, in-hospital mortality, and their determinants.

**Methods:**

Health facility-based prospective observational study was used for three consecutive months among adult patients hospitalized for the surgical case. Socio-demographic, clinical history, medication history, in-hospital complications, and overall treatment outcomes were collected from the medical charts’ of the patients, using a checklist from the day of admission to discharge. PLOS is defined as hospital stay > 75th percentile (≥33 days for the current study). To identify predictor variables for both PLOS and in-hospital mortality, multivariate logistic regression was performed at *p*-value < 0.05 using SPSS version 20. Written informed consent was sought and secured.

**Results:**

Of 269 study participants, 91.8% were improved and discharged. PLOS was recorded in 25.3%; at least 33 days of hospital stay. Overall in-hospital mortality was 4.8%; which is equal to an incidence rate of 0.00193 per person-days, 5.2% in-hospital sepsis, and 2.6% of Hospital-acquired pneumonia (HAP), during their hospital stay. After adjusting for other factors; female gender (*p* = 0.003), emergency admission (*p* = 0.015), presence of Poly-pharmacy (*p* = 0.017), and presence of sepsis (*p* = 0.006) were found to be independent predictors for in-hospital mortality. On top of this, female gender (*p* = 0.026), patients who was paid by government (*p* = 0.007), burn-related surgery (*p* = 0.049), presence of cancer (*p* = 0.027), > 2 antibiotic exposure (*p* < 0.0001), and waiting for surgery for > 7 days (p < 0.0001) were independent predictors for PLOS.

**Conclusion:**

In-hospital mortality rate was almost comparable to reports from developing countries, though it was higher than the developed countries. However, the length of hospital stay was extremely higher than that of reports from other parts of the world. Besides, different socio-demographic, health facility’s and patients’ clinical conditions (baseline and in-hospital complications) were identified as independent predictors for both in-hospital mortality and PLOS. Therefore, the clinician and stakeholders have to emphasize to avoid the modifiable factors to reduce in-hospital mortality and PLOS in the study area; to improve the quality of surgical care.

## Introduction

There was no consistent cutoff point for prolonged length of hospital stay (PLOS). However, it was defined as the total bed-days occupied by a patient during hospitalization for more than the expected length of stay for a certain procedure [1]. The length of hospital stay is often used as a measure of the quality of care and efficient resource utilization; as patients with PLOS extremely account for the consumption of more hospital resources, hospital-acquired infection (HAI), more complications, high mortality rate, denies critically ill patients timely access to treatment and contributing to capacity shortage [1–3].

Nevertheless, PLOS alone is imperfect indicators for the quality of surgical care [4]. Thus, information related to the length of hospital stay, postoperative complications, number of HAI, and mortality is paramount to evaluate efficiency and quality of surgical care [1–4]. As a result of high mortality and complication rates of major surgical procedures in low and middle-income countries (LMICs), surgical safety should be a global concern [5].

Admission with surgery case by itself is found to be one of the causes that contribute to PLOS in LMICs [1]. This, in turn, contributes significantly to extra cost, morbidity, and mortality [1, 6, 7]. Therefore, knowledge of the factors contributing to PLOS and mortality among patients treated with the surgery is an important factor to be considered as a quality of surgical care indicators [8]. Worldwide, 4.8 billion people devoid of access to safe and quality surgical care, due to lack of infrastructure, equipment, and personnel, especially in LMICs [9]. Thus, among patients undergoing surgery, the mortality rates were higher for LMICs [10, 11], for instance, the postoperative death was 2times higher than the global deaths in Africa [12].

Similar to other countries, Ethiopia has been striving to meet the gaps in surgical care, through innovative methods for surgical system development with Saving Lives through Safe Surgery; designed to improve access to safe, essential and emergency surgical and anesthesia care across all levels of the healthcare system [13]. Thus, to early identify the area that needs focus, and for rational allocation and use of the available resources in the country, assessing the surrogate indicators of quality of surgical care such as PLOS, postoperative complications, number of HAI and mortality rate is mandatory. Besides, for the Hospital and stakeholders to take action, the critical step is identifying the status of these quality indicators ahead of preparing for the action plan. However, a study was lacking regarding the predictors of PLOS and in-hospital mortality in the surgical wards of Ethiopia [11]. Even, in developed countries, there were few reports of predictors’ of mortality and PLOS with heterogeneous study results, study population, and type/discipline of surgery with retrospective study design [1]. Therefore, this study aimed to assess treatment outcome, length of hospital stay, in-hospital mortality, and their predictors among adult patients undergoing the surgical procedure.

## Methods and participants

### Study area, period and design

The study was conducted from April 24 to July 24/2017 at Jimma University Medical Center (JUMC). The Surgery department has been run by 8 seniors, 43 residents, 5 general practitioners, and medical interns as rotation [14]. Health facility-based prospective observational study was used. Study populations were patients who were admitted to the surgical ward with either elective or emergency admission during the study period with inclusion criteria.

### Inclusion and exclusion criteria

Patients admitted at the surgical ward at the time of data collection but, did not undergo a surgical procedure at the time of initial data collection and ages of ≥18 years were included. Those who were not willing to participate, patients only on a topical antibiotic for superficial wound care and infected or non-infected burn wound, a trauma-related wound that needs the only debridement or wound care were excluded. Also, patients who were referred or left against medical advice (LAMA) ahead of surgery were excluded from the final analysis.

### Study variables

#### Dependent variables

In-hospital mortality and length of hospital stay.

#### Independent variables

Age, sex, residence, Surgical site infection, types of surgery, type of wound classification, American society of anesthesiology (ASA) class, co-morbid conditions and types, smoking status, appropriateness of surgical antimicrobial prophylaxis (SAP) use, length of hospital stay before surgery, duration of operation, Charlson co-morbidity Index, HAI, sepsis, blood loss during operation, type of surgical admission (emergency or elective), diagnosis and poly-pharmacy.

### Sample size and sampling technique

Sample size (n) was calculated by using a single population proportion formula [15], to determine the minimum sample size required for estimation of true proportion as follow:

1 $$ n1=Z{\left(\partial /2\right)}^2\ast \frac{P\left(1-P\right)}{W^2}={1.96}^2\ast \frac{\left(0.94\ast 0.06\right)}{0.05^2}=87\  for\ mortality\ rate $$

Where; P is the proportion of mortality rate of 0.06 from reference [16], Z is level of confidence = 1.96 with 95% CI, N is the size of the population that the sample is to represent = 1265 per 3 months, W is the margin of error = 5%; Since N is less than 10,000 correction formula was applied [15]:

2 $$ nf=\frac{n}{1+\frac{n}{N}}=81, then\ 5\% non- response\ rate\ was\ added=85 $$

Surveillance by using consecutive type of sampling technique, data was collected from 269 patients available within 3 months to increase its robustness.

### Data collection instrument, process, and management

Semi-structured questionnaires (English version) were used with a slight modification of the tool used by the previously published research [14]. These questionnaires contain five parts, part I (socio-demographic characteristics), part II (patient’s clinical information and Charlson co-morbidity index), part III (patient’s medication information), and Part IV (appropriateness of SAP use) and part V (overall treatment outcome) of the study participants.

Initially, the patient’s chart was reviewed for inclusion and exclusion criteria at the time of admission. Then the 4 data collectors (2 post-graduate clinical pharmacists and 2 surgical nurses) collected information using a pretested checklist. The Centers for Disease Control and Prevention (CDC) wound class was first classified by attending physician and rechecked by one senior surgery resident independently and the discrepancy was solved by communicating with the attending physician. It was done only for the primary surgical procedure, not for the 2nd or 3rd [14]. Then the data collectors reviewed and filled patient’s information from the notes of anesthesia and medical (physicians’ orders and nurses’ notes) and patient interviews (only consent and socio-demographic information). Case finding: overall treatment outcome (hospital-acquired pneumonia/ HAP, sepsis, in-hospital mortality, and length of hospital stay) of patients were measured prospectively.

### Data quality assurance, processing, and statistical analysis

Data were coded, checked for consistency and entered into EpiData manager® version 4. Then the data was exported to SPSS version 20 for analysis. Percentage and frequency were used for categorical variables. Continuous variables were expressed using measures of central tendency and variability such as mean and standard deviation. The model fitness for the variables was evaluated by the Hosmer-Lemeshow goodness of fit test and the *p*-value was found to be 0.245 in binary analysis and *p* > 0.05 for all variables in multivariate analysis. Variables with a significance level of *P* < 0.25 in binary logistic regression were selected as a candidate for multivariate analysis, then significance level was considered at a *p* value of < 0.05.

### Ethical consideration and participants’ consent

The letter of allowance to proceed with the study was sought under protocol number IHRPGQ/103/207 from Jimma University, Institute of Health. The patients were requested for written informed consent. The confidentiality of the patients’ was secured. PLOS is defined as the proportion of the study participants with hospital stay greater than 75th percentile (in JUMC it was found to be ≥33 days) for the entire study population [1].

## Results

### Socio-demographic characteristics of the study participants

Two hundred sixty-nine (operated) patients were included in the study. The mean (± SD) age of the participant was 41.95 ± 17.76 and the majority of the study participants were male (66.5%), and earn less or equal to 6000 Ethiopian birrs (90.7%). Regarding the cost of health care coverage, the majority (82.9%) were classified as a self payer (Table 1).

**Table 1:** Socio-demographic characteristics of the study participants at JUMC, Ethiopia (*N* = 269)

Variables	Categories	Frequency	Percentage
**Age (year)**	mean age ± SD	41.95 ± 17.76 (18 to 90)	
**Sex**	Male	179	66.5
	Female	90	33.5
**Monthly income**	no constant income	47	17.5
	< 1500	95	35.3
	1500–6000	102	37.9
	> 6000	25	9.3
**Health cost coverage**^**a**^	Self-payment	223	82.9
	Free (government)	46	17.1

^a^ - the majority (21.3%) of patients with no constant income were paid by the government or got free service at JUMC

### Clinical characteristics of the study participants

Of the total (269), 40.9% of the study participants had a co-morbid condition. Of which 59 (21.9%) had a chronic type of co-morbidity, 46 (17.1%) oncologic related condition, 48 (17.8%) trauma-related condition, and 14 (5.2%) burn-related surgery. The most common type of surgical discipline carried out at the study area was related to the upper and lower gastrointestinal tract (30.5%) followed by skin and deep tissue (incision, drainage, local excision) (23.0%). The mean ± SD hospital stay before surgery, after surgery and overall hospital stay, was 7.74 ± 14.51, 17.61 ± 16.87 and 25.35 ± 21.42 days respectively, as well as the majority (59.9%) of the patients stayed for at least 15 days in the hospital (Table 2).

**Table 2:** Clinical characteristics and in-hospital complication of the study participants at JUMC, Ethiopia (*N* = 269)

Variables	Categories	Frequency	Percentage
**Co-morbid condition**	Yes	110	40.9
	No	159	59.1
**Number of co-morbidity**	No co-morbid	159	59.1
	1–2 co-morbid	98	36.4
	≥ 3 co-morbid	12	4.5
**Types of co-morbidity**	Non-chronic disease	210	78.1
	Chronic disease	59	21.9
**Oncologic condition**	No	223	82.9
	Yes	46	17.1
**Trauma-related condition**	No	221	82.2
	Yes	48	17.8
**Burn related surgery/skin graft**^**a**^	No	255	94.8
	Yes	14	5.2
**Types of surgical discipline**	upper and lower GIT	82	30.5
	Breast	6	2.2
	Urology	41	15.2
	Cardiothoracic	10	3.7
	Billary tract	5	1.9
	head and neck	40	14.9
	skin and deep tissue (incision, drainage, local excision)	62	23.0
	Other^b^	23	8.6
**The overall length of hospital stay**	≤7 days	35	13.0
	8 to 15 days	73	27.1
	> 15 days	161	59.9
	Mean ± SD (minim- maximum)	25.35 ± 21.42 (1 to 127 days)	
**After operation length of hospital stay**	≤7 days	68	25.3
	> 7 to ≤15 days	101	37.5
	> 15 days	100	37.2
	Mean ± SD (minim- maximum)	17.61 ± 16.87 (0 to 120 days)	
**Before operation length of hospital stay**	≤7 days	198	73.6
	> 7 to ≤15 days	27	10.0
	> 15 days	44	16.4
	Mean ± SD (minim- maximum)	7.74 ± 14.51 (0 to 112 days)	
**Mortality rate**	The incidence rate of death per person-days	0.00193 per person-days	
**In-hospital sepsis**	Incidence rate per person-days	0.00208 per person-days	
**HAP**	Incidence rate per person-days	0.00104 per person-days	

N.B: GIT- gastrointestinal tract, HAP- Hospital-acquired pneumonia, ^a^ was more in female (7.8%) than male (3.9%), ^b^- varicose vein, Hematoma, Gangrene, compartment syndrome, Contracture, and septic arthritis.

### Incidence of hospital-acquired infection (sepsis & HAP) and overall clinical outcome of the study participants

**Fig. 1:**
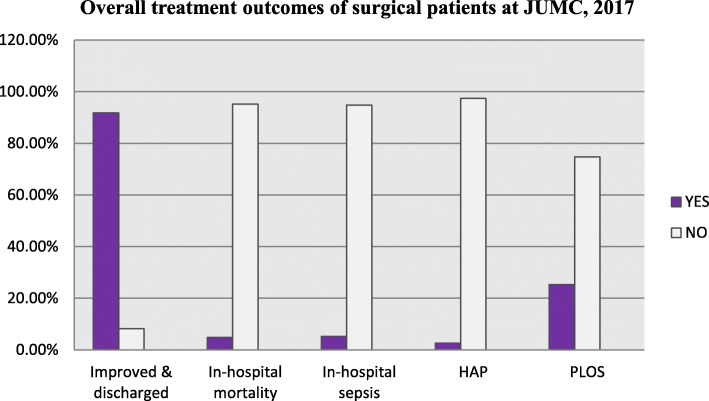
Overall treatment outcomes of patients admitted at the surgical ward of JUMC, Ethiopia (*N* = 269). N.B: PLOS- prolonged length of hospital stay (≥33 days), HAP- Hospital acquired pneumonia

Overall clinical out-come of study, participants were improved in about (91.8%) of cases followed by death in Hospital in about 13(4.8%) of cases. Among the study participants, 5.2%; which is equal to a sepsis incidence rate of 0.00208 per person-days or 2.08 per 1000 person-days and 2.6% Hospital-acquired pneumonia (HAP); which is equal to an incidence rate of 0.00104 per person-days or 1.04 per 1000 person-days developed sepsis and HAP respectively, during their hospital stay. Besides, even though the majority of the patient’s treatment outcome was good (improved), the mortality rate was also high (4.8%); which is equal to an incidence rate of 0.00193 per person-days or 1.93 mortality rate per 1000 person-days. The prolonged length of overall hospital stay (PLOS) was 25.3%; at least 33 days of hospital stay (Table 2 and Fig. 1).

### Predictors of in-hospital mortality and prolonged length of hospital stay (PLOS)

Knowing the predictors of in-hospital death and PLOS is essential in reducing patient-related harm. In turn, it allows us to give special attention to the hope of improving surgery-related treatment outcomes. Hence, the factors that were candidate for multivariate analysis; (*p* value < 0.25) were: gender of female (*p* value = 0.036), ASA class III-IV (*p* value of 0.191), Co-morbid condition (*p* value = 0.007), emergency type of admission (p value = 0.008), CDC wound class II-IV (p value = 0.134), poly pharmacy status (*p* value = 0.001), Amount of blood loss during surgery (*p* = 0.187), Right selection +dose+ timing + duration + route of SAP (*p* value = 0.025), total antibiotic exposure in hospital of > 2 (*p* < 0.015), in-Hospital sepsis in (p value< 0.0001) for in-hospital mortality while, Female gender (*p* < 0.0001), smoking status (*p* = 0.076), health care cost coverage (*p* = 0.023), burn related surgery/ skin graft (*p* = 0.036), presence of cancer (*p* = 0.018), presence of trauma (*p* = 0.060), type of admission (p = 0.023), in-hospital SSI (*p* = 0.035), duration of surgery (*p* = 0.083), appropriate use of SAP (*p* = 0.105), total number of antibiotic exposure (*p* < 0.0001), ASA class (*p* = 0.193), CDC wound class (p = 0 .047), before surgery LOS (days) (p < 0.0001), and number of co-morbidity (*p* = 0.248) for PLOS among study participants (Tables 3 and 4).

**Table 3:** Chi-square test for in-hospital mortality among patients admitted to the surgery ward of JUMC, Ethiopia

Predictor Variables	Categories	The outcome variable (in-hospital death)		*p*-value
		No (%)	Yes (%)	
**Sex**	Male	174 (97.2)	5 (2.8)	0.036
	Female	82 (91.1)	8 (8.9)	
**Age**	age of 18 to 40	142 (94.0)	9 (6.0)	0.329
	age of > 40	114 (96.6)	4 (3.4)	
**Residence**	Rural	143 (96.0)	6 (4.0)	0.492
	Urban	113 (94.2)	7 (5.8)	
**Smoking status**	No	226 (95.4)	11 (4.6)	0.658
	Yes	30 (93.8)	2 (6.2)	
**ASA class**	ASA class I-II	198 (96.1)	8 (3.9)	0.191
	ASA class III-IV	58 (92.1)	5 (7.9)	
**Co-morbid condition**	No	156 (98.1)	3 (1.9)	0.007
	Yes	100 (90.9)	10 (9.1)	
**type of admission**	Elective	136 (98.6)	2 (1.4)	0.008
	Emergency	120 (91.6)	11 (8.4)	
**CDC wound class**	class I	48 (100)	0 (0)	0.134
	class II-IV	208 (94.1)	13 (5.9)	
**Poly-pharmacy status**	No	221 (97.4)	6 (2.6)	0.001
	Yes	35 (83.3)	7 (16.7)	
**Charlson CI**	CCI of 0 (no co-morbid)	91 (93.8)	6 (6.2)	1.00
	CCI of > 1 (yes co-morbid)	165 (95.9	7 (4.1)	
**Amount of blood loss during surgery**	< 1500 ml	150 (96.8)	5 (3.2)	0.187
	≥1500 ml	3 (75.0)	1 (25.0)	
	unknown (not recorded)	103 (93.6)	7 (6.4)	
**Overall LOS**	≤ 7 days	37 (94.9)	2 (5.1)	1.00
	> 7 days	219 (95.2)	11 (4.8)	
**After surgery LOS**	≤ 7 days	65 (95.6)	3 (4.4)	1.00
	> 7 days	191 (95.0)	10 (5.0)	
**Before surgery LOS**	≥7 days	187 (94.4)	11 (5.6)	0.524
	> 7 days	69 (97.2)	2 (2.8)	
**duration of surgery**	≤1 h	81 (95.3)	4 (4.7)	0.994
	> 1 h	133 (95.0)	7 (5.0)	
	Not recorded	42 (95.5)	2 (4.5)	
**Right selection + dose + timing + duration + route of SAP**	All criteria correct	47 (88.7)	6 (11.3)	0.025
	At least one criteria not correct	209 (96.8)	7 (3.2)	
**Total number of antibiotic exposure**	≤ 2 antibiotic exposure	200 (97.1)	6 (2.9)	0.015
	> 2 antibiotic exposure	56 (88.9)	7 (11.1)	
**HAP**	No	251 (95.8)	11 (4.2)	0.329
	Yes	5 (71.4)	2 (28.6)	
**Sepsis**	No	247 (96.9)	8 (3.1)	< 0.0001
	Yes	9 (64.3)	5 (35.7)	
**SSI**	No	213 (95.1)	11 (4.9)	1.00
	Yes	43 (95.6)	2 (4.4)	

*LOS* length of hospital stay, *HAP* hospital-acquired pneumonia, *SAP* surgical antimicrobial prophylaxis

**Table 4:** Bivariate analysis for PLOS among patients admitted to the surgical ward of JUMC, Ethiopia

Predictor Variables	Categories	The outcome variable (PLOS) > 75th percentile (≥33 days)		**p-value	*p-value
		No (%)	Yes (%)		
**Sex**	Male	146 (81.6)	33 (18.4)	0.169	0.000
	Female	55 (61.1)	35 (38.9)		
**Place of residence**	Rural	110 (73.8)	39 (26.2)	0.547	0.706
	Urban	91 (75.8)	29 (24.2)		
**Level of monthly income**	no constant income	33 (70.2)	14 (29.8)	0.291	0.396
	< 1500	67 (70.5)	28 (29.5)		
	1500–6000	81 (79.4)	21 (20.6)		
	> 6000	20 (80.0)	5 (20.0)		
**Smoking status**	No	173 (73.0)	64 (27.0)	0.522	0.076
	Yes	28 (87.5)	4 (12.5)		
**Health care cost coverage**	Self	171 (76.7)	52 (23.3)	0.023	0.103
	Free (gov’t)	30 (65.2)	16 (34.8)		
**Co-morbid condition**	No	117 (73.6)	42 (26.4)	0.443	0.606
	Yes	84 (76.4)	26 (23.6)		
**Burn-related surgery**	No	194 (76.1)	61 (23.9)	0.043	0.036
	Yes	7 (50)	7 (50)		
**Presence of cancer**	No	173 (77.6)	50 (22.4)	0.028	0.018
	Yes	28 (60.9)	18 (39.1)		
**Presence of trauma**	No	160 (72.4)	61 (27.6)	0.873	0.060
	Yes	41 (85.4)	7 (14.6)		
**Presence of any chronic disease**	No	154 (73.3)	56 (26.7)	0.221	0.323
	Yes	47 (79.7)	12 (20.3)		
**Type of admission**	Elective	95 (68.8)	43 (31.2)	0.864	0.023
	Emergency	106 (80.9)	25 (19.1)		
**Poly-pharmacy**	No	169 (74.4)	58 (25.6)	0.861	0.811
	Yes	32 (76.2)	10 (23.8)		
**In-hospital SSI**	No	173 (77.2)	51 (22.8)	0.244	0.035
	Yes	28 (62.2)	17 (37.8)		
**In-hospital sepsis**	No	191 (74.9)	64 (25.1)	0.366	0.771
	Yes	10 (71.4)	4 (28.6)		
**HAP**	No	196 (74.8)	66 (25.2)	0.887	1
	Yes	5 (71.4)	2 (28.6)		
**Age (years)**	18 to 40	115 (76.2)	36 (23.8)	0.188	0.539
	> 40	86 (72.9)	32 (27.1)		
**Duration of surgery (hrs.)**	≤ 1	57 (67.1)	28 (32.9)	0.391	0.083
	> 1	107 (76.4)	33 (23.6)	0.678	
	Not recorded	37 (84.1)	7 (15.9)	0.172	
**Appropriate use of SAP**	Yes	35 (66.0)	18 (34.0)	0.404	0.105
	No	166 (76.9)	50 (23.1)		
**Total number of antibiotic exposure**	≤ 2 antibiotic exposure	165 (80.1)	41 (19.9)	0.017	0.000
	> 2 antibiotic exposure	36 (57.1)	27 (42.9)		
**ASA class**	I to II	150 (72.8)	56 (27.2)	0.499	0.193
	III to IV	51 (81.0)	12 (19.0)		
**CDC wound class**	I	34 (70.8)	14 (29.2)	0.047	0.494
	II to IV	167 (75.6)	54 (24.4)		
**Charlson CI score**	Zero	70 (72.2)	27 (27.8)	0.303	0.469
	> 1	131 (76.2)	41 (23.8)		
**Before surgery LOS (days)**	≤ 7	163 (82.3)	35 (17.7)	0.000	0.000
	> 7	38 (53.5)	33 (46.5)		
**Number of co-morbidity**	Zero	117 (73.6)	42 (26.4)		0.626
	1 to 2	76 (77.6)	22 (22.4)	0.248	
	≥ 3	8 (66.7)	4 (33.3)	0.248	

*p value of chi-square, **p-value for binary logistic regression analysis

**Fig. 2:**
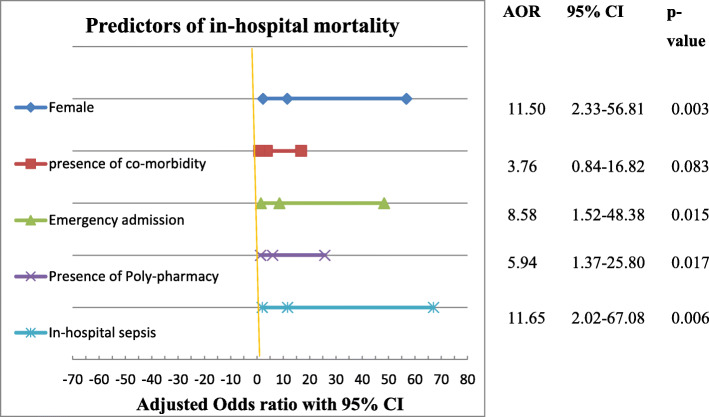
Forest plot describing the result of multivariate analysis for in-hospital mortality among patients admitted to the surgical ward of JUMC, Ethiopia. N.B: AOR- Adjusted odds ratio, CI- Confidence interval

A stepwise backward multivariate logistic regression analysis showed that, study participants with female gender were about 12 times more likely to have a negative outcome (death) relative to male gender [AOR = 11.50 (2.33–56.81) at 95% CI; p value = 0.003]. Emergency surgery increase the probability of in-hospital death by about 9 times than elective surgery [AOR = 8.58 (1.52–48.38) at 95% CI; *p* value = 0.015]. Presence of Poly-pharmacy and in-hospital sepsis increase the risk of death by about 6 times [AOR = 5.94 (1.37–25.80) at 95% CI; p value = 0.017 and 12 times [AOR = 11.65 (2.02–67.08) at 95% CI; *p* value = 0.006], respectively than patients who devoid of poly-pharmacy and sepsis (Fig. 2).

**Fig. 3:**
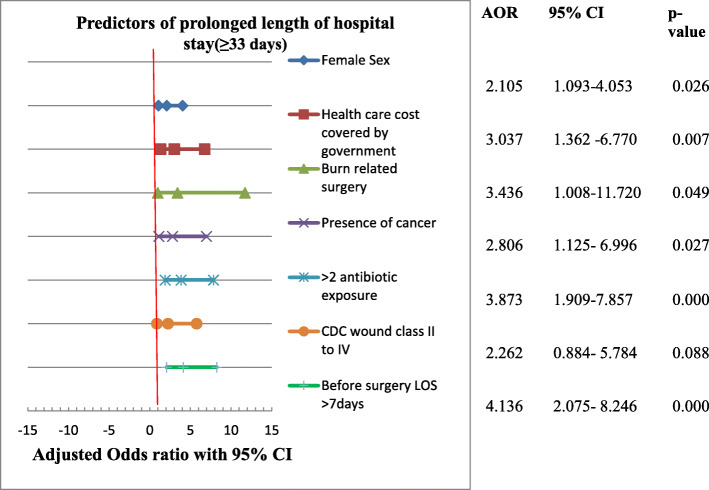
Forest plot describing the result of multivariate analysis for PLOS among patients admitted to the surgical ward of JUMC, Ethiopia. N.B: LOS- length of hospital stay, PLOS- Prolonged length of hospital stay, AOR-Adjusted odds ratio, 95% C.I for multivariate logistic regression

On the other hand, patients with female gender were 2 times more likely to have PLOS [AOR = 2.105 (1.093–4.053) at 95% C.I; p = 0.026], patients who was paid by government were 3 times more likely to have PLOS [AOR = 3.037 (1.362–6.770) at 95% C.I; p = 0.007], burn related surgery was about 3 times more likely to increase risk of PLOS [AOR = 3.436 (1008–11.720) at 95% C.I; p = 0.049], and presence of cancer increases the likelihood of PLOS by about 3 times [AOR = 2806 (1.125–6.996) at 95% C.I; p = 0.027]. Besides, >two antibiotics exposure during hospital stay increases the chance of PLOS by about 4 times [AOR = 3.873 (1.909–7.857) at 95% C.I; p < 0.0001], and awaiting for surgery for > 7 days increases the probability of PLOS by about 4 times [AOR = 4.136 (2.075–8.246) at 95% C.I; p < 0.0001] relative to their counterpart for each of variables respectively (Fig. 3).

## Discussion

This was a 3-month health facility-based prospective observational study to assess treatment outcome (in-hospital mortality & PLOS with 75th percentile or ≥ 33 days) and their predictors among adult patients at the surgery ward of JUMC had found a high rate of in-hospital mortality and PLOS among hospitalized patients. The socio-demographic and clinical characteristics (baseline and in-hospital complications were found to be independent predictors for both in-hospital death and PLOS for 269 patients included in the study.

Among the study participant, 5.2% in-hospital sepsis incidence rate and 2.6% HAP were identified during their Hospital stay in JUMC. The postoperative HAP in JUMC is lower than the study done on intra-abdominal surgical patients (10.7%) and increased risk of mortality by 10 fold [17]. The reason for this difference might be that patients with gastro-intestinal surgery are more prone to aspiration pneumonia and high rate of HAP, especially gastrostomy even than other gastro-intestinal surgery (decreases gastric acid then increase bacterial colony thus, increase risk of HAP). The surgical injury/ procedure is one factor that increases the risk of HAI (HAP, sepsis, or septic shock) which in turn increases the risk of in-hospital death among patients undergoing surgery [17, 18]. Another study added postoperative complications occurred in 18·2%; infection was the most common complication and 2·1% of mortality rate [12]. Therefore, the hospital and department of surgery of JUMC should take action to reduce prolonged hospitalization as well as a HAP like bloodstream infection or sepsis and HAP.

Regarding treatment outcome in terms of hospital stay, the mean overall hospital stay was 25.06 ± 21.415 days and in-hospital mortality was 13 (4.8%). However, hospital stay was higher, compared to study from Gondar, a median of 14.2 ± 8.4 days, also, the median hospital stay was 4 days in African Hospitals [4], and 6 days median stay [2]. Additionally, in Singapore [19] median hospital stay was 7 days which was extremely lower than JUMC; which needs a revision of health care service at the study area.

while the clinical outcome was almost comparable with a study from Gonder, Ethiopia; improved (90.4%) and died (6.0%) [16], but, less than the study done in Tikur Anbessa Specialized Hospital; 7.0% overall death per procedure [11]. In St Paul General Specialized Hospital and Gondar University Hospital, Ethiopia after the onset of symptoms, delay of acute abdomen and abdominal trauma for more than 3 days before operation resulted in 67% death [20]. Other studies also reported in-hospital death rate of 3.4% [8], 2.2% [21] however, the in-hospital mortality rate of 14.7% [22] was higher than our study result. The reason for this difference was because the later study included older patients (> 80 years unlike that of > 18 years). On the other hand, the mortality rate was reported from 2.3 to 48.5% for patients undergoing a different type of surgery and heterogeneous study population as well as different severity [8, 23–26]; indeed the death rate is high for low-income countries because of different reason [10, 12], for instance, in America, the overall mortality rate was 0.28% [27].

PLOS might be due to prolonged waiting for surgery (mean of 7.74 ± 14.51 days), as a result of a low number of the senior surgeon and complicated case referrals from other Hospitals; which will contribute for overall PLOS. This is similar to another study report from the low-income country; low physician-to-case ratio [5]. The second reason might be due to the difference in disease prevalence like burn patients contributed to longer hospitalization. This should gate attention from stakeholders because reducing hospitalization can reduce extra cost, risk of HAI, and increase productivity as a result of early discharge.

On the other hand, female gender, emergency admission, presence of Poly-pharmacy, and the presence of sepsis or in-hospital bloodstream infection were found to be independent predictors of in-hospital death at JUMC. Other studies showed that After adjusting for all co-morbidities, female gender [20, 28] while in contrast male gender [11, 21] and emergency surgery [8, 10, 11, 21], development of in-hospital complications [7, 17, 18, 22], sepsis [25] was an independent predictor for in-hospital mortality [8, 28], but, there was no clear reason for the gender-related increase in mortality. The gender-related discrepancy in predicting mortality might be due to the difference in baseline disease or type of operation (general surgery- coronary artery bypass graft surgery- thoracic surgery) respectively. The reason for emergency surgery related to more death is attributable to a complicated case and less time for preoperative care. Besides, the poly-pharmacy related more mortality is attributable to multiple diseases and/ or complicated cases that might directly or indirectly contribute to death.

Even though there is no consistent definition of prolonged length of hospital stay (greater than median LOS [3], 50th [4], 75th & 90th [2] or 95th [1] percentile was used in different studies), it is usually used as a measure of quality indicators and efficient resource utilization [1, 3], yet imperfect proxy for quality [4]. It is common for surgical patients to have PLOS because of different reasons [1]. This issue was true for surgical patients admitted in JUMC; PLOS was 25.3% with 75th percentile; at least 33 days of stay. Even though PLOS definition is different 50th vs. 75th percentile, PLOS was in line with another study (27.7%) had a hospital stay of at least 8 days [4]. In contrary, ours is higher than PLOS of 23.1% with 95th percentile but, the cut of point is almost similar to our study; at least 34 days of stay [1] and the 75th and 90th percentile length of stay (LOS) were 9 and 16 days, respectively which is almost less by half [2] than that of JUMC. Because developing countries have many obstacles, which hinder quality surgical care [9].

In addition, female gender, Health care cost coverage by government, burn-related surgery, presence of cancer, > 2 total number of antibiotic exposure, and > 7 days waiting for surgery were the independent predictors for PLOS among the study participants. In contrast to our study finding, the male gender was found as independent predictors for PLOS [1]. There was no clear reason for gender as a predictor of PLOS but, the reason for female being as independent predictors in our case was as a result of burn-related surgery contributed for a longer stay for awaiting graft for a longer period than other elective surgery. Again patients with burn-related surgery were female patients (as evidenced by our data 3.9% male versus 7.8% female (Table 2)); who pass their time near the fire for cooking. There were different predictors for PLOS which again contributed to in-hospital complications like HAI (sepsis, SSI) [2] and PLOS was contributed for 3 fold in-hospital mortality [1], on top of this preoperative patient and health facility-related factors are the independent predictors for PLOS [3]. There was no clear reason why patients whose health care cost covered by the government stay more in hospital than self payer at JUMC. This might be those patients are economically disadvantaged, for example, if the same medication for operation is ordered and unavailable in the hospital Pharmacy, they have to wait till medication is available (especially for elective cases) because they cannot buy from private Pharmacy (Table 1). This result was in line with other studies where low socio-economic status is independent predictors for PLOS [1].

The limitation of this study was that the use of consecutive sampling techniques (non-probability) than that of the probability sampling technique, owing to the cost for data collectors to get the total patients within a short period. Second, being single-center study. We hope future research will solve this issue.

## Conclusion and recommendations

Even though the mortality rate at JUMC is within an acceptable range relative to study from a developing country, yet it was higher compared to the studies from developed countries. The prolonged length of hospital stay (PLOS ≥33 days) was extremely higher than the previous reports. Besides these, the study also revealed a high rate of hospital-acquired infection (sepsis and HAP) during the study period, which has been contributing to in-hospital mortality. After adjusting for other variables, female gender, emergency surgery, presence of Poly-pharmacy, and presence of in-hospital sepsis were independent predictors for in-hospital mortality. Besides, female gender, Health care cost coverage by government, burn-related surgery, presence of cancer, > 2 total number of antibiotic exposure, and > 7 days waiting for surgery were the independent predictors for PLOS.

Therefore, in the study area, sepsis warrants great attention to improve patients’ treatment outcomes or reduce in-hospital mortality. Based on the independent predictors for PLOS, patient’s safety and quality of care could be improved by decreasing the waiting time for surgery, and other modifiable factors. This may increase productivity, access to surgery, and reduce person-bed-day as a result of early discharge.

## Data Availability

The datasets used and/or analysed during the current study are available from the corresponding author on reasonable request.

## Abbreviations

ASA: American Society of Anesthesiology
CCI: Charlson co-morbidity index
CDC: The Centers for Disease Control and Prevention
HAI: Hospital-acquired infection
HAP: Hospital-acquired pneumonia
JUMC: Jimma University Medical Center
LMIC: Low and middle-income country
PLOS: Prolonged length of hospital stay
SAP: Surgical Antimicrobial Prophylaxis
SSI: surgical site infection
